# Celecoxib decreases growth and angiogenesis and promotes apoptosis in a tumor cell line resistant to chemotherapy

**DOI:** 10.1186/0717-6287-47-27

**Published:** 2014-06-16

**Authors:** Carlos Rosas, Mariana Sinning, Arturo Ferreira, Marcela Fuenzalida, David Lemus

**Affiliations:** Programa Disciplinario de Anatomía y Biología del Desarrollo, Instituto de Ciencias Biomédicas, Facultad de Medicina, Universidad de Chile, Santiago, Chile; Departamento de Neurología y Neurocirugía, Hospital Clínico Universidad de Chile, Santiago, Chile; Programa Disciplinario de Inmunología, Instituto de Ciencias Biomédicas, Facultad de Medicina, Universidad de Chile, Santiago, Chile

**Keywords:** Angiogenesis, Celecoxib, Tumor

## Abstract

**Background:**

During the last few years it has been shown in several laboratories that Celecoxib (Cx), a non-steroidal anti-inflammatory agent (NSAID) normally used for pain and arthritis, mediates antitumor and antiangiogenic effects. However, the effects of this drug on a tumor cell line resistant to chemotherapeutical drugs used in cancer have not been described.

Herein we evaluate the angiogenic and antitumor effects of Cx in the development of a drug-resistant mammary adenocarcinoma tumor (TA3-MTXR).

**Results:**

Cx reduces angiogenesis in the chick embryonic chorioallantoic membrane assay (CAM), inhibits the growth and microvascular density of the murine TA3-MTXR tumor, reduces microvascular density of tumor metastases, promotes apoptosis and reduces vascular endothelial growth factor (VEGF) production and cell proliferation in the tumor.

**Conclusion:**

The antiangiogenic and antitumor Cx effects correlate with its activity on other tumor cell lines, suggesting that Prostaglandins (PGs) and VEGF production are involved. These results open the possibility of using Celecoxib combined with other experimental therapies, ideally aiming to get synergic effects.

## Background

Non-steroidal anti-inflammatory drugs (NSAIDs) is a heterogeneous group of drugs associated with inhibition of the inflammation process, mainly targeting enzymes such as cyclooxygenase (COX), involved in the synthesis of prostaglandins (PG) from arachidonic acid. However, NSAIDs have been related to COX-2 and COX-1 inhibition, considering that COX-1 inhibition may cause gastrointestinal bleeding and ulcers, and COX-2 inhibition is associated to anti-inflammatory, antipyretic and analgesic effects.

COX-2 has not been only related to inflammation but also angiogenesis, proliferation and tumor growth. There is evidence of an overexpression of COX-2 in a variety of cancers [1–3]. Patients over-expressing COX-2 in pancreatic tumor cells have a worse prognosis than those who do not [4, 5].

Celecoxib (Cx) is a selective COX-2 inhibitor approved by the Food and Drug Administration (FDA) for rheumatoid arthritis, osteoarthritis and acute pain, but in the last years it has been proposed as an agent that can intervene signal transduction pathways associated with COX-2 expression and increase the levels of endogenous inhibitors of angiogenesis, called endostatins [6, 7]. Moreover, NSAIDs decrease tumor progression for some malignancies such as colon cancer [8–10]. For this reason, Cx has been proposed for the treatment of colon, pancreatic, and breast cancer, suppressing angiogenesis and promoting apoptosis [5, 10, 11]. Finally Cx inhibits the growth of a meningioma *in vivo*, decreases COX-2 activity and lowers PG concentrations and angiogenesis, promoting higher rates of apoptosis [4].

Considering these results altogether, Cx has been related to antitumoral and antiangiogenic effects. Konturek et al. [12] proposed that PG-E bind to EP receptor mediates apoptosis evasion, angiogenesis, proliferation and migration. Moreover, PG-E modulates survivin and VEGF levels, which are associated to evasion of apoptosis and angiogenesis respectively. With this proposal, COX-2 inhibition mediated by Cx could reduce tumor growth, angiogenesis and promote apoptosis, through a reduced PG production. This effect is relevant in acquired resistance to conventional therapy such as chemotherapy, because Cx effect is independent of the chemotherapy action mechanism. For this reason, we hypothesize that Cx reduces angiogenesis and tumor growth in a mammary tumor cell line resistant to chemotherapy such as TA3-MTXR. Previously, we have shown that Cx at 1000 ppm reduces liver metastasis but not lung metastasis, in mice with a multiresistant adenocarcinoma TA3 [13]. On the other hand, Sulindac, a NSAID that inhibits COX-1 and COX-2 activity, reduces angiogenesis in an *in vivo* model [14]. Herein we evaluate the angiogenic and antitumor effects of Cx in the development of a drug-resistant mammary adenocarcinoma tumor.

## Results

### Cx reduces microvascular density in the chick-CAM assay

**Figure 1:**
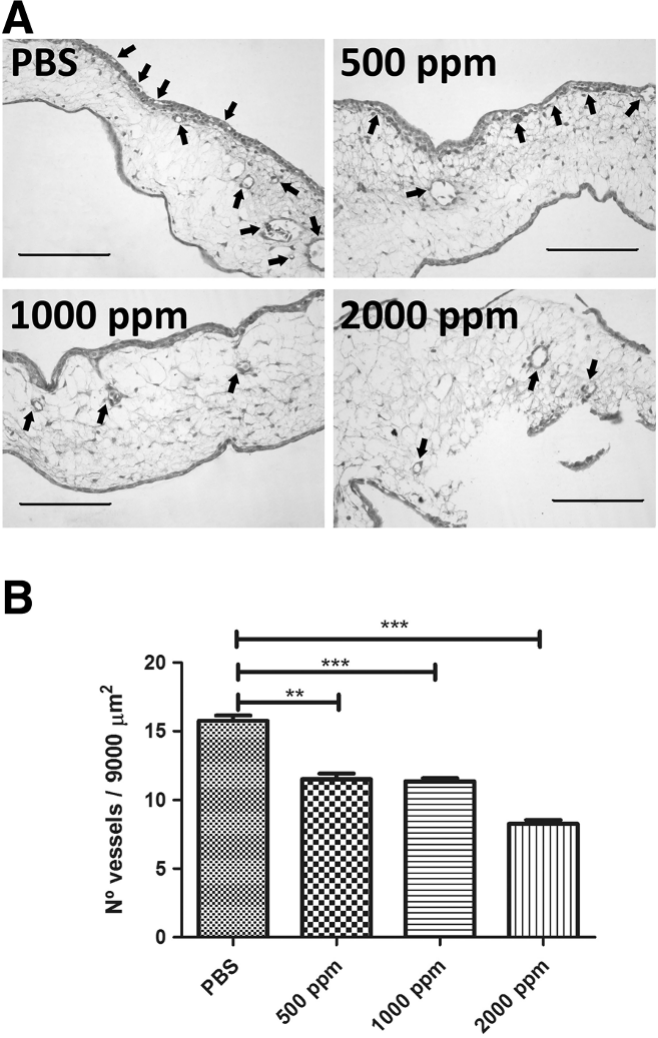
**Cx reduces microvascular density in the chick-CAM assay.** PBS (Control), Cx at 500, 1000 and 2000 ppm was instilled on chick chorioallantoic membranes (CAM) as described in Methods. Membranes were fixed and stained with H&E-Alcian Blue. Membranes were visualized **(A)** and number of vessels per 9000 μm^2^ was counted **(B)**. Blood vessels are represented by arrows. (**: p < 0.001; ***: p < 0.0001). Scale bar: 100 μm.

The potential antiangiogenic effect of Cx in CAM assay was assessed when 500–2000 ppm of Cx were assayed. PBS was used as negative control. The chick CAM is a vascular membrane formed by two mesodermal layers, allantois and chorionic epithelium. Transversal sections of CAM showed a chorionic epithelium with small blood vessels, a mesenchyme with medium-size blood vessels and allantoic epithelium. Morphologically, tissue sections treated with Cx at 500 and 1000 ppm showed a normal morphology without apparent damage. However, Cx used at 2000 ppm, induced tissue alterations, mainly epithelial destruction (Figure 1A).

Microvascular density analysis showed that number of vessels/9000 μm^2^ on CAM was 15.76 ± 0.38 for PBS, 11.50 ± 0.40 for Cx 500 ppm, 11.36 ± 0.22 for Cx 1000 ppm and 8.27 ± 0.28 for Cx 2000 ppm.A strong antiangiogenic effect was observed with Cx used at 500 (p < 0.001), 1000 (p < 0.0001) and 2000 ppm (p < 0.0001) when is compared with PBS group (Figure 1B).

### Cx inhibits the growth of a murine A/J mammary tumor (TA3-MTXR)

**Figure 2:**
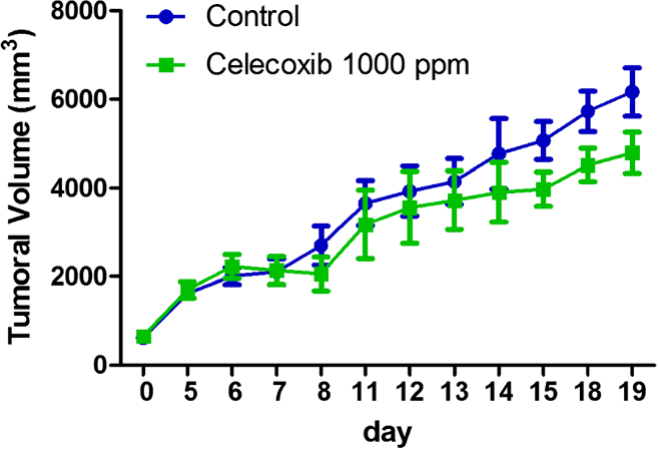
**Cx inhibits the growth of a murine A/J mammary tumor.** 900000 tumor cells were inoculated s.c. in the flank of A/J female mice. Cx treatment and measurement of tumor development are described in the Methods section. The graph represents the tumor volume of control (n = 6) and Cx-treated (n = 6) mice. Cx showed an anti-tumor effect compared with control (p < 0.05). For bioethical reasons, the experiment was stopped at 19th day. Bars represent standard errors. Wilcoxon signed rank test p < 0.05.

Cx effect on the *in vivo* growth of the TA3-MTXR tumor cell line was assessed. All tumor-injected mice had similar thigh size on day 0. This remained constant until day 6, where an increase in tumor volume could be observed and measured. From this moment on, treated group (n = 6) began receiving oral treatment with Cx at 1000 ppm, with inhibitory effects (p < 0.05) on tumor growth when compared with control group (n = 6) (Figure 2).

At the 15th day, the control group showed a volume of 5069 ± 427.1 mm^3^ and the treated group had a volume of 3978 ± 385.2 mm^3^. The treated group presented a statistical significant difference when it was compared with control group (p = 0.0435).

At the 18th day, a statistically significant difference was detected when the treated group (4519 ± 376.6 mm^3^) was compared with the control group (5727 ± 455.8 mm^3^) (p = 0.0341).

At the 19th day, the control group presented a volume of 6168 ± 550 mm^3^ while the treated group had a volume of 4790 ± 463 mm^3^. These results confirm that Cx promoted a 22.3% reduction in tumor volume (p = 0.0422). For bioethical rules, assay was stopped at 19th day, because, one mouse of control group died.

### Cx inhibits microvascular density of a murine A/J mammary tumor (TA3-MTXR)

**Figure 3:**
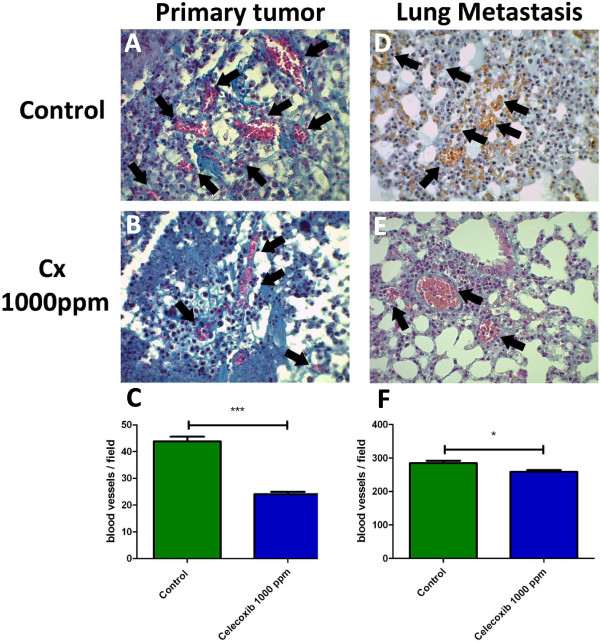
**Cx inhibits microvascular density of a murine A/J mammary tumor (TA3-MTXR).** Histological sections stained with Arteta were visualized at 400× in an optical microscopy and blood vessels were counted. Left panel shows a representative section of untreated primary tumor **(A)** or Cx-treated **(B)**. Blood vessels were counted **(C)** and a statistical significance was found (***: p < 0.0001). Right panel shows a representative section of untreated lung metastasis **(D)** or Cx-treated **(E)**. Blood vessels were counted **(F)** and a statistical significance was found (*: p = 0.0031). Blood vessels are represented by black arrows.

Histological sections of tumor and lungs were stained with Arteta for improving blood vessels visualization. The tumor sections presented a peripheral and a central zone. The Peripheral zone is an area with fibroblasts, collagenous fibers and blood vessels. The Central zone showed necrotic areas, tumor cell pleomorphism and disorganized small blood vessels, typical architecture of tumor angiogenesis. The group inoculated only with the TA3-MTXR tumor (Figure 3A) averaged 43.8 ± 1.74 vessels/field. On the other hand, the group treated with Cx at 1000 ppm presented 24.1 ± 0.86 vessels/field (Figure 3B). A significant decrease in the number of vessels per area was detected in the treated group (p < 0.0001) (Figure 3C).Lung sections showed normal parenchyma surrounding a group of tumor cells in a “cannon ball” shape. This formation had tumor cells, neoangiogenic vessels and a loss of lung architecture. In lung sections, the control group (Figure 3D) averaged 284.8 ± 7.21 vessels/field, while Cx (1000 ppm) the treated animals (Figure 3E) averaged 258.7 ± 5.65 vessels/field, (p = 0.0031) (Figure 3F).

### Cx decreases proliferation of a murine A/J mammary tumor (TA3-MTXR)

**Figure 4:**
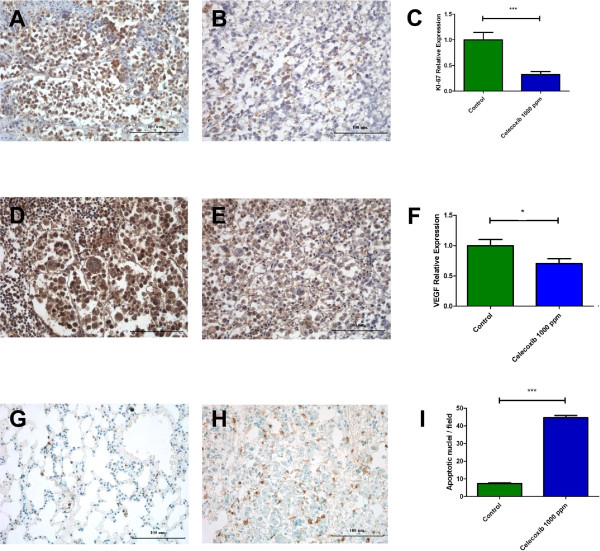
**Cx decreases proliferation, VEGF production and promotes apoptosis of a murine A/J mammary tumor (TA3 MTXR).** Tumor samples showed a decreased proliferation in Cx-treated group **(B)**, when compared with control group **(A)**. Relative expression is represented **(C)** (***: p < 0.0001) VEGF expression was reduced in treated group **(E)** when it was compared with control group **(D)**. Relative expression is represented **(F)** (*: p = 0.0178). Apoptotic nuclei of lung sections were labeled with a TUNEL assay and treated group **(H)** showed an increased apoptotic nuclei/field when it was compared with control group **(G)**. Apoptotic nuclei per field are represented **(I)** (***: p < 0.0001).

Histological sections of tumor were incubated with a Rabbit Polyclonal Anti – Human Ki-67 antibody, a protein associated to cell proliferation. In the control group, immunomarked cells (Ki-67 positive) were tumor cells, even when they are surrounded by lymphocytes and polymorphonuclear cells (Figure 4A). Quantification of immunomarked cells in tumor showed that the control group had a Ki-67 relative expression of 1.00 ± 0.14 while the Cx-treated group showed a lower Ki-67 relative expression (0.322 ± 0.060). Low quantities of immunomarked cells were found in this group, but all of this were tumor cells (Figure 4B). Treated group presented a statistically significant difference when it was compared with control group (p < 0.0001) (Figure 4C).

### Cx decreases VEGF production of a murine A/J mammary tumor (TA3-MTXR)

Histological sections of tumor were incubated with a Anti-VEGF_165_ Polyclonal Antibody, which detected the main protein associated to angiogenesis.The control group showed a moderate to abundant presence of VEGF with a cytoplasmic staining pattern of tumor cells. Quantification of VEGF expression was 1.00 ± 0.100 (Figure 4D). In contrast, the Cx-treated group showed only a mild to moderate VEGF presence in tumor with a relative expression of 0.70 ± 0.08 (Figure 4E). The Cx-treated group showed a statistically significant difference when it was compared with control group (p = 0.0178) (Figure 4F).

### Cx promotes apoptosis of a murine A/J mammary tumor (TA3-MTXR)

Histological sections of lung metastasis were assessed with a TUNEL Assay for fragmented DNA detection, a characteristic condition of apoptosis. We observed 7.30 ± 0.39 and 44.6 ± 1.24 apoptotic nuclei/field in lungs from the control (Figure 4G) and Cx-treated (Figure 4H) groups, respectively (p < 0.0001) (Figure 4I). We also observed that, in a primary tumor, apoptosis increased (p < 0.05) (not shown) after treatment with Cx at 1000 ppm.

## Discussion

We propose that Cx decreases growth in a drug resistant mammary adenocarcinoma tumor. Moreover, Cx decreases angiogenesis and promotes apoptosis in the same tumor cell line.

Cx inhibits microvascular density in the CAM assay at different concentrations. This result was useful to define the optimal concentration in a tumor (Figure 1). Previous reports demonstrated that COX-2 inhibitors suppressed angiogenesis on CAM [15]. However, the COX-2 inhibitor assessed was nimesulide at 100 μmol/L. In our study we demonstrated that Celecoxib suppressed angiogenesis at 500 ppm and 1000 ppm. Higher doses than 2000 ppm induces tissue destruction.Cx inhibits tumor growth in a murine A/J mammary adenocarcinoma tumor (Figure 2). When Cx has been used in a particular tumor cell line, results are rather contradictory among different authors.

Raut et al. [16] achieved a reduction in the L3.6pl pancreatic tumor line impact at only very low concentrations (10 mg/kg) of Cx injections.

Ragel et al. [4] showed that Cx at 1500 ppm orally administered reduced the growth of the tumor line IOMM-Lee.

Klenke et al. [17] used injections of Cx in a 30 mg/kg concentration, obtaining a decrease of an A549 lung tumor, between days 21 and 28.

Xu et al. [5] used Panc-1 tumor cell line and observed a decrease in tumor volume and weight when oral Cx was used at 1500 ppm.

Harris et al. [18] and Jang et al. [19] generated a tumor using orally administration of DMBA and 1500 ppm of Cx for treatment. Only the first group obtained a significant decrease in tumor volume. Dai et al. [20] demonstrated that Celecoxib 1000 mg/kg inhibited rat carcinogenesis and cancer development.

Although results and doses may vary for each tumor cell line, our study demonstrated that oral administration of Cx at 1000 ppm reduced tumor growth in the TA3-MTXR line. Moreover, since 15th to 19th day there was a significant difference between both groups. It is important to consider the number of animals. In our study, twelve mice divided in two groups were used. Although the number of animals was low, the results were significant. Our results propose that Cx reduces angiogenesis and proliferation and promotes apoptosis, as reflected in a reduction of tumor growth. However, this effect does not seem to prevent metastasis from primary tumor. Previously, we have shown that lung metastasis do not decrease in the Cx-treated group [13]. Accordingly with this result, Cx could not reduce proliferation even when Cx reduces microvascular density in pulmonary metastases, but we do not make a comparison between flank tumor and metastasis. This proposal needs to be clarified in further investigations.

Our results show that Cx inhibits microvascular density of a murine A/J mammary tumor and lung metastasis. The microvascular density comparison succeeded in establishing that there were significant differences in the tumor and lung when treated with Cx, reducing vascular density (Figure 3C, F). Other organs like the spleen and heart did not differ because they were not invaded by tumor cells (data not shown). The process of tumor angiogenesis is associated with the formation of new blood vessels, which are tortuous, small and abundant in areas of hypoxia. Since angiogenic microvessels generated by pro-angiogenic factors are diminished by the action of Cx, the results are concordant with previous reports correlating angiogenesis and COX-2 activity [21].

Our immunohistochemical study showed that Cx caused a decrease in the presence of KI-67 (Figure 4C), VEGF (Figure 4F) and increased presence of apoptotic nuclei (Figure 4I) in TA3-MTXR tumor cells. These effects on the tumor can be explained based on previous studies. Thus, Ghosh et al. [22] reported that COX-2 activity might activate carcinogens. Moreover, PGE_2_, the major downstream effector of COX-2 is associated to apoptosis inhibition, cell adhesion, tumor growth and promotes angiogenesis. On the other hand, Tarnawski and Jones [23] and Rüegg [24] proposed that COX-2 is an enzyme that synthetize PGs, and their overexpression and/or PGs production participate actively in the development of angiogenesis and apoptosis inhibition. With this effects, Cx as a COX-2 inhibitor might inhibit angiogenesis, reduces tumor growth and promotes apoptosis. In our study, Cx inhibits tumor growth, microvascular density and promote apoptosis of tumor cells resistant to chemotherapy.

Kerbel [25] described the signaling pathway of VEGF, a potent proangiogenic factor that produces tumor cells and promotes survival, proliferation and migration of endothelial cells, critical steps involved in angiogenesis. Our results showed that Cx reduces VEGF production of a murine mammary tumor. Moreover, in some organs where metastasis occurred, such as the lung, VEGF production was abundant; especially in certain outlying areas where tumor cells form nodules but Cx treatment reduced VEGF levels in that area (data not shown). The idea that VEGF is reduced with the use of Cx is supported by Kim et al. [26]; Rodríguez et al. [27] and Vaish and Sanyal [28] who defined a relation of β-catenin with COX-2 and survivin.

Brandao et al. [29] reported that short-term COX-2 inhibition by Cx inhibits proliferation reflected by a reduction of Ki-67 positive cells in patients with breast cancer. In our study, Cx at 1000 ppm decreases proliferation of a murine mammary tumor resistant to chemotherapy using the same marker. The association between COX-2 activity and proliferation has been previously proposed. Wu et al. [30] demonstrated that Cx inhibits proliferation and induces apoptosis via PGE_2_ pathway.

Jendrossek [31] proposed that the pro-apoptotic effect of Cx is not only mediated by COX-2 inhibition. Cx affects apoptotic signaling at multiple levels such as decreasing expression levels of Mcl-1 and survivin. Moreover, apoptosis induction by Cx may not depend on the presence of COX-2. Our results demonstrate that Cx promotes apoptosis of murine mammary tumor.

Konturek et al. [12] describes that the binding of PGs to its receptor promotes evasion of apoptosis through increased survivin and Bcl-2. Moreover, the same binding might induce VEGF expression through hypoxia inducible factor 1 alpha (HIF-1α), and cell proliferation and migration via MAPK. These mechanisms explain at least in part, the association between VEGF and COX-2/PGs.

The association between PGs and tumorigenesis is confirmed by Wang and Dubois [32] who showed that PGs are involved in processes of proliferation and survival, angiogenesis and migration. Moreover, Dai et al. [20] and Brandao et al. [29] showed that Celecoxib inhibits the proliferation of breast cancer.

Cx-treated group results might be explained at least in part by the effect of Cx on PGs synthesis. 6 days after inoculation, an obvious but significantly smaller tumor was developed. These tumor cells produced low levels of VEGF, because COX-2 action and PG production was much lower. Although proliferation and VEGF production was significantly decreased, it was not completely reduced, because tumor cells can produce VEGF through other mechanisms, both genetic and epigenetic. Our results reinforce the idea that Cx reduces angiogenesis, proliferation and promotes apoptosis, probably through a diminished PGs production.

Previous reports of antitumor activity of Cx were assessed in many tumor cell lines. However it has been poorly investigated the role of Cx in a drug resistant tumor cell line. The TA3-MTXR cells come from a mammary murine carcinoma tumor line of ascitic growth, resistant to many chemotherapeutical drugs, but mainly to methotrexate (MTX), an antifolate which prevents the optimal availability of folic acid by the cells, preventing DNA synthesis and concomitant inability to replicate [33]. This resistance to MTX does not affect the action mechanism of Cx, which occurs through completely different pathways. This can be extrapolated to the clinical management of patients with tumors resistant to MTX treatment, where they can receive Cx treatment and reduce tumor progression [34, 35]. On the other hand, Khan et al. [36] showed that Cx and continuous low-dose of cyclophosphamide and MTX provides a little anticancer effect.

These results reinforce the idea of Cx use in the cancer treatment either free or through Cx-loaded nanoparticles [37]. On the other hand, it is necessary to explore a combined treatment between Cx and other drugs [38, 39]. For instance, a new approach could involve *T. cruzi* Calreticulin (TcCRT), or derived domains that inhibit angiogenesis, in several experimental set ups, and in very low concentrations [40, 41].

## Conclusions

Cx reduces tumor growth in a concentration of 1000 ppm, decreases microvascular density in tumor and metastatic organs, reduces the presence of VEGF and promotes apoptosis of multiresistant TA3 tumor cells. The antiangiogenic and antitumor Cx effects correlate with its activity on other tumor cell lines, suggesting that Prostaglandins (PGs) and VEGF production are involved. Cx could be used alone or combined with other antitumor molecules, ideally aiming to get synergic effects. For now, Cx is used for some cancer types. However, future investigations may clarify whether this drug alone or combined is effective in clinical situations where there is resistance to MTX.

## Methods

### Animal welfare

Eight week old adult (20–25 g) female A/J strain mice (n = 12) (*Mus musculus*) were obtained from our Central Animal Facility Central Vivarium. Experimental protocols were approved by the Bioethics Committee, Faculty of Medicine, University of Chile.

### Tumor growth assay

The effect of 1000 ppm of Cx on *in vivo* growth of the TA3-MTXR murine mammary tumor cell line was assessed as described previously [42]. Briefly, TA3-MTXR cells come from a mammary murine carcinoma tumor cell line of ascitic growth. Methotrexate (MTX) resistance was performed by weekly passages of ascitic fluid from mice combined with increasing concentrations of MTX (0.1 to 2.5 mg/kg/48 hrs) until the appropriate resistance. Twelve mice were inoculated in the right lower limb area, at day 0 with 900,000 tumor cells. At day 6, mice were orally treated with 1000 ppm of Cx (n = 6) or water as control (n = 6). Width and length of the tumor was measured with a digital caliper. The volume was then calculated as previously described [43]. Tumor growth was assessed until 19th day where mice were euthanized for obtaining tumor and organs samples for histological procedures. For bioethical rules the experiment need to be stopped at 19th day.

The experiments were validated by using the Wilcoxon Signed Rank test (Graph Pad Prism 5). P values < 0.05 were considered as statistically significant.

### Drug preparation

Cx (Pfizer Laboratories) was diluted in water at 500, 1000 and 2000 ppm. 1000 ppm concentration was used in drinking water, as described previously [4].

### Immunohistochemistry

Tumor and lung metastasis from the primary tumor were obtained at 19th day and then fixed in a 10% buffered formalin solution for 48 hours. Serial sections of 5 μm were obtained. In order to evaluate cell proliferation, a Rabbit Polyclonal Anti – Human Ki-67 antibody (1:500) (Novocastra, Newcastle Upon Tyne, UK) was used. Briefly, Ki-67 is a nuclear antigen associated to cellular proliferation. The polyclonal antibody (Novocastra, Cat#NCL-Ki67-P) binds to Ki-67 antigen in the granular components of the nucleolus during late G1, S, G2 and M phases [44]. To detect Vascular Endothelial Growth Factor (VEGF), an Anti-VEGF_165_ Polyclonal Antibody (1:100) (Millipore, CA, USA) was used and then revealed by the “HistoMouse-MAX Kit” (Invitrogen, Camarillo, USA) which is based on the use of a secondary antibody conjugated with horseradish peroxidase and subsequently revealed with 3,3′-diaminobenzidine. Relative Expression was assessed with 30 microscopic fields and analyzed by Image J Software (NIH, USA). The average standard error was then calculated and applied to the t-student test.

### Evaluation of apoptosis

To evaluate DNA fragmentation (an indicator of apoptosis), the *FragEL*™ *DNA Fragmentation Detection Kit* (Calbiochem, Darmstadt, Germany) was used. This system is based on labeling fragments of DNA of apoptotic cells by using a TUNEL assay. Histological sections of tumor and lung metastasis obtained at 19th day were assessed and apoptotic nuclei were counted in light microscope.

### Microvascular density quantification

To count of blood vessels were counted at 400× in histological sections from tumors and lungs obtained at 19th day and were stained with Arteta, as described previously [44].

### Chick chorioallantoic membrane (CAM) assay

The CAM assay was performed as described [40]. Briefly, 40 fertilized White Leghorn hen eggs (National Public Health Institute, Santiago, Chile) were used. The eggs were incubated for 48 h in a humid 38.5°C atmosphere. After extracting 2–3 ml of albumin, a small window was opened in the egg, in order to allow separation of the CAM from the shell during the embryo development. The window was temporarily sealed with adherent tape and the eggs were incubated for additional 5 days. Then, sterile methyl cellulose discs (5 mm diameter, 0.25 μm pore size, 125 μm thickness) (Advantec MFS Inc., CA, USA), were deposited on the CAMs. Immediately, 10 μl of Cx at 500, 1000 or 2000 ppm were directly added onto the filters. The windows were then sealed and the eggs were incubated for additional 72 h, as indicated above. Then, the CAMs were sliced, following the filters contours, and fixed in 10% (v/v) formaldehyde. Tissue sections were prepared by standard procedures for conventional light microscopy, which aimed to detect mainly acid polysaccharides, nuclei and cytoskeleton. Blood vessels were counted in a light microscope with a 1 cm^2^ micrometric grid, divided in 1 mm^2^ sections. Ten of these sections, corresponding to a tissue area of 9000 μm^2^ were counted. Blood vessels were identified by their endothelial cells and red blood cells in their lumens. Counting, carried out in a double-blind fashion, was performed in 35 microscopic fields of CAM tissue segments, adjacent to the filter edge. One way-ANOVA with Dunnett’s Multiple Comparison Test was used to assess the statistical significance, with a confidence interval of 99%.
